# Lethal Interpersonal Violence in the Middle Pleistocene

**DOI:** 10.1371/journal.pone.0126589

**Published:** 2015-05-27

**Authors:** Nohemi Sala, Juan Luis Arsuaga, Ana Pantoja-Pérez, Adrián Pablos, Ignacio Martínez, Rolf M. Quam, Asier Gómez-Olivencia, José María Bermúdez de Castro, Eudald Carbonell

**Affiliations:** 1 Centro Mixto UCM-ISCIII de Evolución y Comportamiento Humanos, Madrid, Spain; 2 Departamento de Paleontología, Facultad Ciencias Geológicas, Universidad Complutense de Madrid, Madrid, Spain; 3 Área de Antropología Física, Departamento de Ciencias de la Vida, Universidad de Alcalá, Madrid, Spain; 4 Department of Anthropology, Binghamton University (SUNY), Binghamton, New York, United States of America; 5 Division of Anthropology, American Museum of Natural History, New York, New York, United States of America; 6 Departamento Estratigrafía y Paleontología, Facultad de Ciencia y Tecnología, Euskal Herriko Unibertsitatea, Bilbao, Spain; 7 IKERBASQUE, Basque Foundation for Science, Bilbao, Spain; 8 Équipe de Paléontologie Humaine, Département de Préhistoire, Muséum national d'Histoire naturelle, Musée de l’Homme, Paris, France; 9 Centro Nacional de Investigación sobre la Evolución Humana, Burgos, Spain; 10 Àrea de Prehistòria, Departamento d’Història i Història de l’Art, Universidad Rovira i Virgili, Tarragona, Spain; 11 Institut Català de Paleoecologia Humana i Evolució Social, Tarragona, Spain; 12 Institute of Vertebrate Paleontology and Paleoanthropology of Beijing, Beijing, China; University of Hawaii at Manoa, UNITED STATES

## Abstract

Evidence of interpersonal violence has been documented previously in Pleistocene members of the genus *Homo*, but only very rarely has this been posited as the possible manner of death. Here we report the earliest evidence of lethal interpersonal violence in the hominin fossil record. Cranium 17 recovered from the Sima de los Huesos Middle Pleistocene site shows two clear perimortem depression fractures on the frontal bone, interpreted as being produced by two episodes of localized blunt force trauma. The type of injuries, their location, the strong similarity of the fractures in shape and size, and the different orientations and implied trajectories of the two fractures suggest they were produced with the same object in face-to-face interpersonal conflict. Given that either of the two traumatic events was likely lethal, the presence of multiple blows implies an intention to kill. This finding shows that the lethal interpersonal violence is an ancient human behavior and has important implications for the accumulation of bodies at the site, supporting an anthropic origin.

## Introduction

Interpersonal violence (lethal and nonlethal) in prehistory is of special interest since it provides a window into human social relations in the past and may be associated with subsistence contexts such as competition for scarce resources, population density or territorial defense [1–2]. Interpersonal violence can be manifested in different ways in the archaeological record, including trauma on hominin bones, which makes it susceptible to approach these questions in paleoanthropological contexts through the application of modern forensic methods of trauma analysis. Interpersonal violence is well-documented since at least Neolithic times [3–5]. In recent prehistory, perimortem human manipulation in the form of cutmarks or bone breakage patterns has often been interpreted as cannibalism [6–9] and could indicate violence between human social groups [10]. Evidence of cannibalism and defleshing is also present during the Paleolithic and has been documented in fossil hominins dating to at least the Early Pleistocene [11–14]. Nevertheless, there is no evidence of direct traumatic injury as a possible cause of death in any of these Pleistocene cases.

Cranial and postcranial trauma are relatively common among Middle and Upper Pleistocene hominins and in most cases show signs of healing (S1 Table), indicating survival of the individual [15–18]. Currently, there are only two examples in the fossil record that are tentatively considered cases of lethal interpersonal violence. The Shanidar 3 Neandertal shows a penetrating lesion to the left ninth rib consisting of a parallel-sided groove with exostoses along its margins [19]. Nevertheless, some bone remodelling is apparent, suggesting this individual survived for several weeks after the lesion, and it is not clear that the final cause of death was related to the rib injury. The Upper Paleolithic *Homo sapiens* individual Sunghir 1 shows a perimortem sharp trauma in the first thoracic vertebra that has been interpreted as the likely cause of death. While this would seem to represent a relatively clear case of lethal interpersonal violence, the authors did not rule out the possibility of a hunting accident [20]. Here we report on the presence of perimortem lethal cranial traumatic lesions in a Middle Pleistocene individual from the Sima de los Huesos (SH) site, a singular case in the hominin fossil record.

The SH site has yielded an extraordinarily large sample of Middle Pleistocene (c. 430 kya) hominin fossils belonging to the Neandertal clade [21] and corresponding to a minimum of 28 individuals [22]. During the time the hominin bones accumulated at the SH site, the only possible access route to the site were through a deep (13 m) vertical chimney [23]. Given the skeletal part representation in the collection [24], it is likely that entire bodies were deposited at the site. There are no cutmarks on any of the 6700+ hominin bones recovered to date, and carnivore manipulation (tooth marks) of the bones is rare [25]. The origin of the accumulation has been highly debated, and four different hypotheses (carnivore activity, transport by geological agents, accidental falls, and intentional accumulation of bodies by hominins) have been proposed [24,26–29].

**Fig 1:**
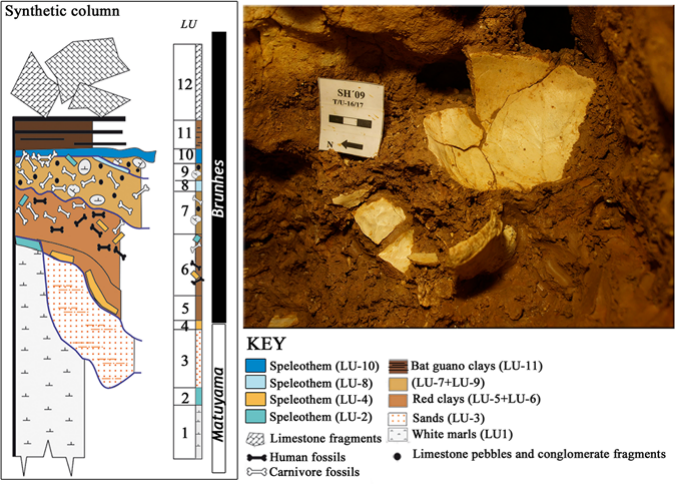
Stratigraphy of the Sima de los Huesos site (modified from Arsuaga et al. [21]). The hominin bones were recovered in Lithostratigraphic Unit 6 (LU-6) dated to c. 430ka [21]. This unit is composed of pure red clays, filtering into the conduit system from overlying soils with little or no lateral transport, and very low velocity of sedimentation (decantation by dripping water) [23]. The figure also shows a detailed image of Cr-17 during its excavation at the site. Note the pure red clay that covers the cranial bones (partially cleaned *in situ* to enhance visualization) and the typical *in situ* postmortem (fossil diagenetic) fractures of the cranial vault. Photo credit: Javier Trueba (Madrid Scientific Films).

Recent taphonomic studies have ruled out carnivores and geological processes as accumulation agents [23,25,30]. The sediment of the hominin deposit (Lithostratigraphic Unit-6, LU-6) is indicative of a low-energy depositional environment (decantation) (Fig 1), and the small size of the SH site suggests only short-distance transport of the fossils within the site [21,23]. Furthermore, the red clay of LU-6 is devoid of extraclasts which provides strong evidence that the fossils were not subjected to long-distance transport, but likely accumulated *in situ* at the SH conduit [23]. The pattern of postcranial fractures in the assemblage indicates that the vast majority occurred after burial, and were caused by the overlying sediment pressure [30]. Nevertheless, a low proportion (around 4%) of postcranial perimortem fractures were found [30] and several cases of antemortem healed cranial injuries are also present in the SH sample [31] (S1 Table).

## Materials and Methods

Cranium 17 (Cr-17) is a very complete specimen composed of 52 bone fragments preserving the complete facial skeleton, including the partial upper dentition (the right C^1^ to M^3^ and the left C^1^ to M^2^), the frontal bone, the sphenoid bone (missing only the body), the left parietal bone, the left temporal bone (lacking only the mastoid process) and most of the occipital bone. The preserved right M^3^ is fully functional and shows only slight wear, indicating that Cr-17 belonged to a young adult individual [21]. Junta de Castilla y León (Burgos, Spain) is the repository of the fossil (Cr-17). All necessary permits (excavation permit granted by the Junta de Castilla y León) were obtained for the described study, which complied with all relevant regulations.

Observations of the fractures were made with a Nikon SMZ800 Stereoscopic light microscope, as well as a DINO-LITE digital microscope. Detailed images of the fractures were made with a Nikon Digital Sight DS-Fi1 camera.

### Contour and trajectory analysis of the traumas

3D imaging provides an opportunity to present critical aspects of cranial blunt force trauma [32], such as shape analysis [33], and to investigate plausible injury trajectories. Cr-17 was CT scanned in the coronal plane using an industrial XYLON MU 2000-CT scanner at the Universidad de Burgos (Spain) with scanner energy of 180 kV and 4 mA. Slice thickness was collimated to 0.5 mm, inter-slice spacing was 0.2 mm, and the approximate field of view was 225 mm. A total of 1108 slices was obtained as a 1024 x 1024 matrix of 32 bit Float format, with a final pixel size of 0.219 x 0.219 mm. A virtual (3D CT) model of the cranium was generated from the resulting slices using the Mimics 16.0 (Materialise N.V.) software package.

Both, fracture angle and cortical delamination were measured on the virtual reconstructions using Mimics 16.0 software tools. Fracture angle is the angle formed by the fracture surface and the bone cortical table, while cortical delamination or bevelling is the cleavage between the diploë and the inner/outer table.

The form of both of the fractures was analyzed in order to compare their contours. Relying on the virtual reconstruction of the cranium, ten equidistant points were placed along the preserved outlines of both traumatic fractures (T1 and T2). The first point was placed on the notch in each fracture, and the 3-D coordinates of all the points were recorded. The coordinates were then transferred to the Morphologika 2.5 software program to perform the superposition of the two outlines relying on the first landmark (the notch) as a reference point. The two fractures outlines were rotated and superimposed but were not rescaled, to permit comparison of the true size of the lesions.

In forensic cases, the impact trajectories are estimated following the vectors of the entrance/exit wounds [34], especially for gunshot wounds. In the present case, the trajectory of the impact for each fracture was established by creating a normal vector to the plane of the fracture defined by the points outlining its contour.

## Results

### SH Cranium-17 injuries

**Fig 2:**
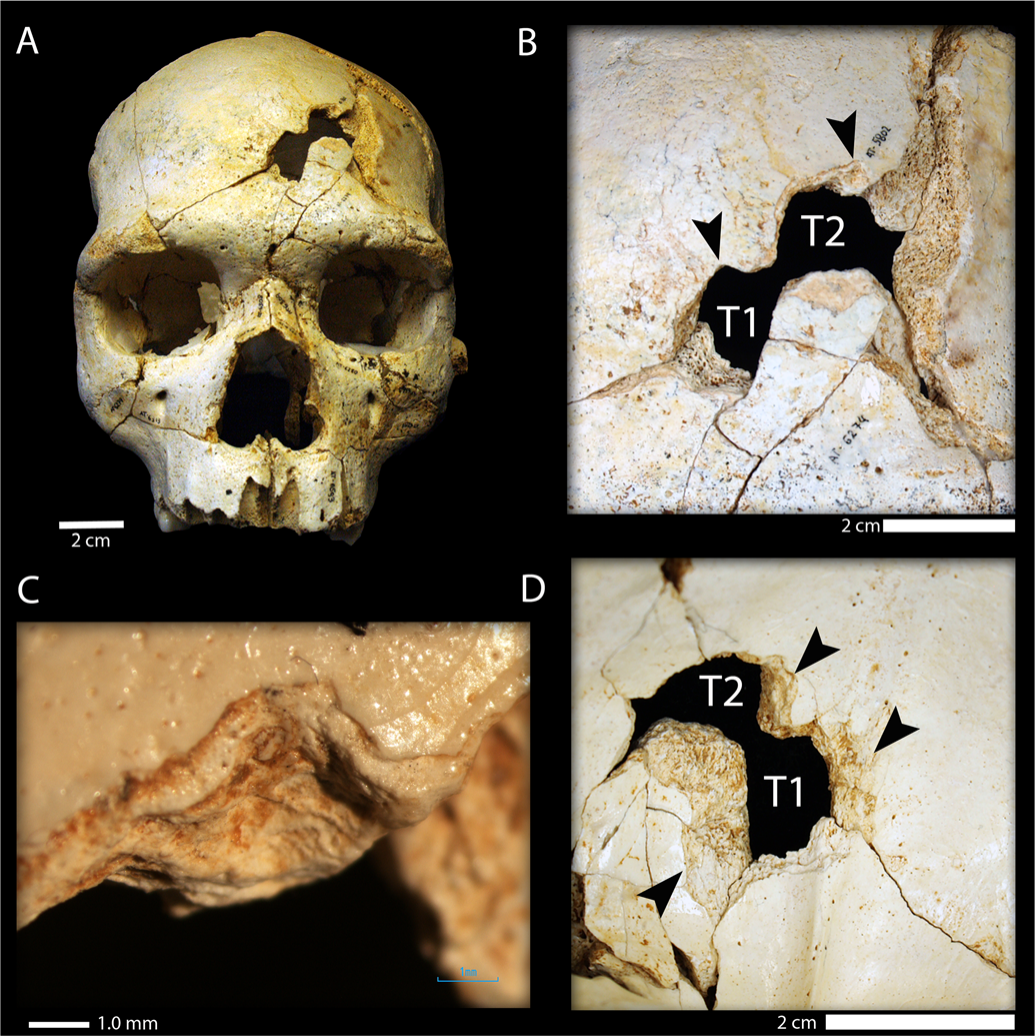
Cranium 17 bone traumatic fractures. (A) Frontal view of Cranium 17 showing the position of the traumatic events T1 (inferior) and T2 (superior); (B) Detailed ectocranial view of the traumatic fractures showing the two similar notches (black arrows) present along the superior border of the fracture outlines. Note that the orientation of the two traumatic events is different; (C) Detail of the notch in T1 under 2X magnification with a light microscope. (D) Endocranial view of T1 and T2 showing the large cortical delamination of the inner table (black arrows).

**Fig 3:**
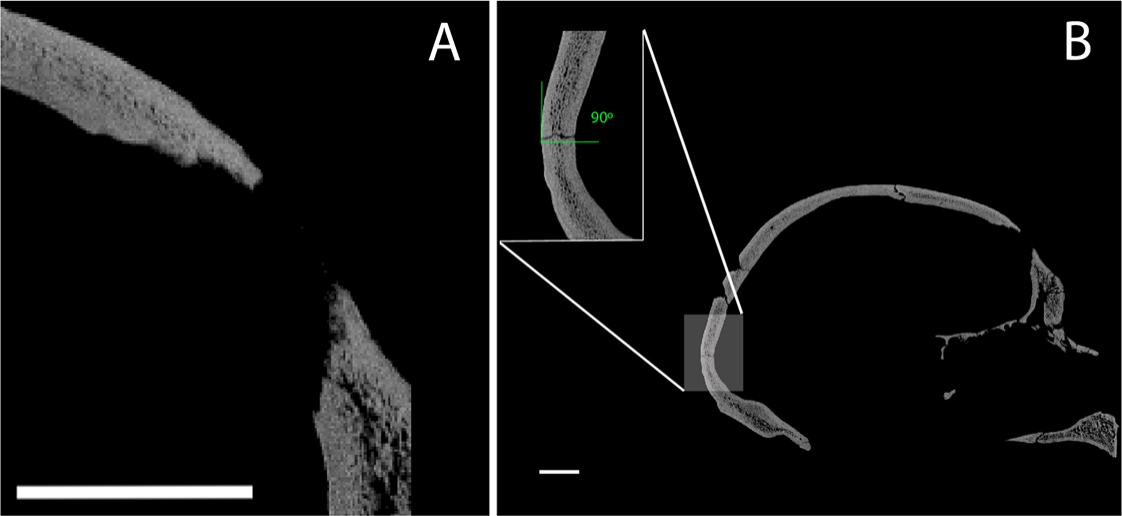
CT cross-sections comparing perimortem and postmortem fractures in the SH crania. (A) Perimortem fracture of the frontal bone in SH Cranium 17; (B) Postmortem fracture in the occipital bone of SH Cranium 17 showing a right angle at the point of breakage. Scale bar = 2 cm.

The 52 fragments that comprise Cr-17 show clear postmortem fractures along their edges (Table 1, Figs 2 and 3) typical of a dry bone breakage pattern commonly found in fossils under diagenetic conditions. No evidence of carnivore manipulation, such as tooth marks, is present on this specimen [25]. On the left side of the frontal squama, this individual shows two sub-rectangular-shaped fractures, Trauma 1 (T1) and Trauma 2 (T2) (Fig 2).

**Table 1:** Fracture properties of SH Cr-17.

Region		Fracture outline			Angle**		Surface		Cortical delamination	
	N	Straight	Curved	Depressed	Mean	SD	Smooth	Jagged	Present	Absent
**Frontal** *	6	5	1	0	95.6	7.6	0	6	0	6
**Occipital**	8	5	3	0	90.3	10.4	0	8	0	8
**Left parietal**	9	8	1	0	100.2	11.8	0	9	0	9
**T1**	1	0	0	1	38.7^†^	-	1	0	1	0
**T2**	1	0	0	1	36.6^†^	-	1	0	1	0

*T1 and T2 excluded.

**Angle between the cortical table and surface of the fracture (in degrees).

^†^ For T1 and T2, two angles were measured on opposite sides of each fracture (T1 = 32.52° and 44.81°; T2: 49.22° and 23.97°).

N = Number of fractures analyzed.

SD = Standard Deviation.

The bone fragments that comprise the Cr-17 vault, as well as in the entire SH neurocranial sample, show a clear dominance of straight/curved fracture outlines, right angles between the cortical table and surface of the fracture, jagged surfaces and absence of cortical delamination along the fracture edges (Table 1). These are typical features of a dry bone breakage pattern, with fractures commonly considered as occurring postmortem in forensic cases (see Table 2) These criteria are applicable to the SH cranial collection, as well as the postcranial bones [30]. The T1 and T2 fractures do not follow this dry bone breakage pattern.

**Table 2:** Perimortem (fresh bone) vs postmortem (dry bone) fracture properties.

Feature	Description	Perimortem features	Postmortem features	Literature
**Fracture texture**	Morphology of the broken bone surface	Smooth*	Jagged	[32,35–40]
**Fracture angle**	Angle between the cortical table and surface of the fracture	Oblique* (acute or obtuse)	Right	[32,35–42]
**Cortical delamination or bevelling**	Cleavage between the diploë and the inner/outer table	Present *	Absent	[35,43]
**Bone flakes**	Small bone fragments attached to the impact site	Present*	Absent	[35,41]

* Present in Cr-17 traumatic fractures

**Fig 4:**
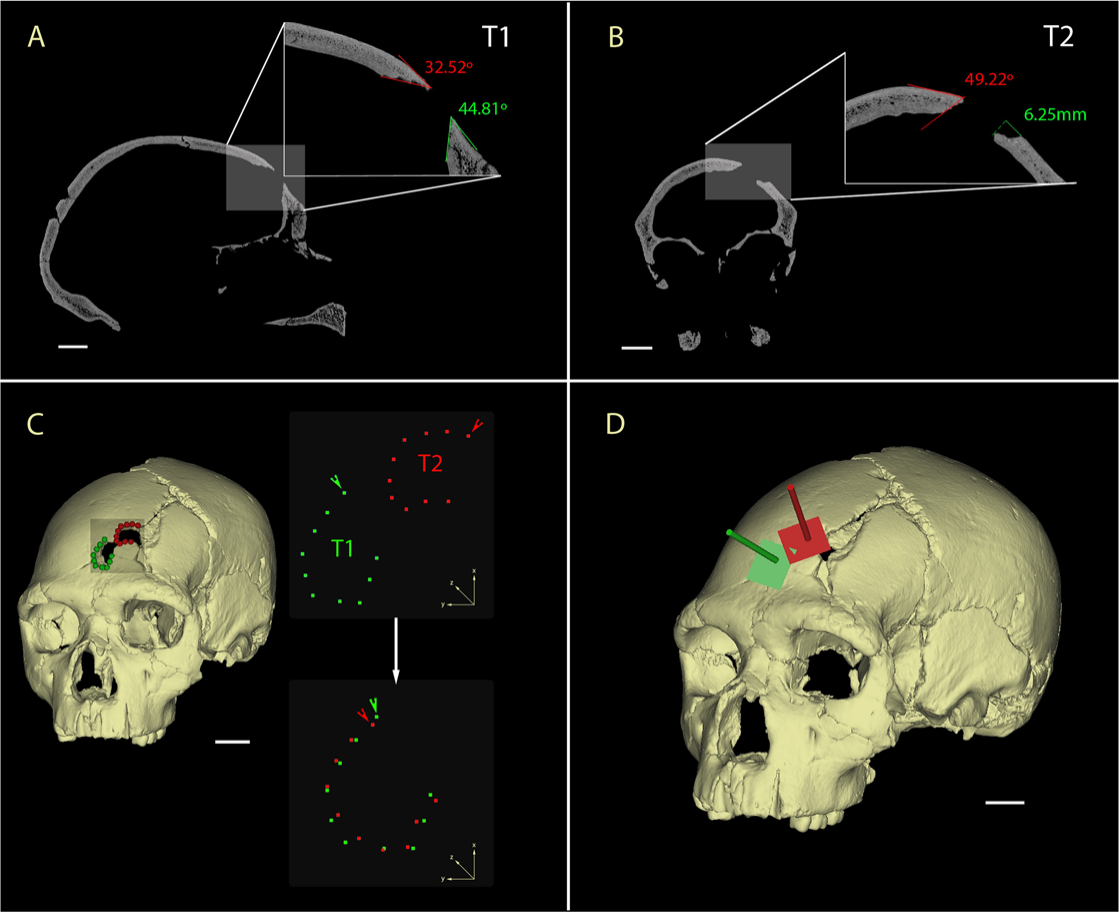
CT analysis of the Cranium 17 traumas. A) Sagittal cross-section of the T1 showing the acute fracture angles (32.5°-44.8°); B) Coronal cross-section of the T2 in detail showing the acute fracture angle (49.2°) and the large cortical delamination of the outer table (6.25mm); C) Virtual reconstruction of the SH Cranium 17 (left) showing the fractures on the left frontal squama and the landmarks used for the shape analysis (blue = T1 and red = T2). 3D landmarks of T1 and T2 in their original position (upper right), with arrows indicating the first landmark placed on the notch. Rotation and superposition of the 3D landmarks of T1 and T2 using the first landmark of the two fractures as reference points (lower right). D) Reconstruction of the trajectory of the impacts relying on the planar orientations of the external outlines of the fractures (blue = T1 and red = T2). Scale bars 2 cm.

Trauma 1 (T1) is located 16 mm lateral of bregma and 72 mm anterior to the coronal suture. Trauma 2 (T2) is located 30 mm lateral of bregma and 56 mm anterior to the coronal suture. Both T1 and T2 are connected and affect the inner and outer bone tables. On the ectocranial surface (Fig 2B), the outline of T1 is sharp, with well-defined borders, and shows fracture lines radiating from the center of the trauma involving both the external and internal tables. The fracture angle of T1 is oblique (32.5°-44.8°, Fig 4). A small, but distinct, notch is present along the superior border of the outline of T1 on the ectocranial surface (Fig 2). On the endocranial surface there is a halo of cortical delamination (Fig 2) that varies from a minimum of about 4.9 mm to a maximum of about 8.8 mm.

As in the case of T1, T2 exhibits an oblique fracture angle between the fracture edge and the external bone table (24°-49.2°, Fig 4), radiating fracture lines, a smooth fracture surface, cortical delamination on the endocranial surface and a small, but distinct, notch along the superior border of the fracture outline. Furthermore, just lateral to T2 on the ectocranial surface, there is a large irregular cortical delamination (13.5 mm in maximum length) that exposes the diploë. The diploë is also exposed along the fracture edges in both T1 and T2. There are no signs of bone remodeling (e.g. presence of woven bone) in any of these exposure areas (Figs 3 and 4).

### Differential Diagnosis

The fracture pattern in both T1 and T2 is characterized by oblique angles, smooth surfaces, and the presence of cortical delamination (Table 1). This is the expected pattern for localized perimortem blunt force trauma [35,39,44] and is consistent with penetration of the endocranial bone table. In contrast to postmortem fractures, perimortem fractures occur while the bone is still fresh (i.e. surrounded by soft tissue and/or preserving the organic matrix), and the fracture properties are well defined (Table 2). Furthermore, the morphology and characteristics of the two fractures in Cr-17 fit the criteria to be defined as depression fractures [1,45,46] that exhibit no evidence of healing (Fig 3). Depression fractures result from a concentration of energy sufficient to cause local failure to the bone and may be characterized as perforating and penetrating fractures with or without associated radial fractures [45,47].

The dimensions and contours of the two depression fractures were found to be almost indistinguishable (Fig 4) (including the presence of a similarly-placed notch in both fracture outlines), strongly suggesting that both fractures were caused by the same object. Furthermore, the fractures show different orientations (T2 is rotated relative to T1, Figs 2 and 4) and different trajectories (Fig 4) implying that each fracture was caused by an independent impact.

## Discussion and Conclusions

Because soft tissue decomposition occurs sometime after the death of the individual, it is possible the injuries in Cr-17 could have been produced either during the free-fall down the vertical shaft (the mode of entry of the hominin cadavers to the site) or inside the SH chamber after the body arrived to the site. The few cases of perimortem fractures in the postcranial remains might be attributable to the corpse landing on a hard object (e.g. limestone block) at the bottom of the vertical shaft [30]. However, in the case of Cr-17, the same object likely produced the two fractures. Thus, any scenario related to the free-fall would require the highly improbable occurrence of the same object striking the skull twice. The same criteria is valid to exclude limestone block-falls inside the SH chamber once the skull was deposited in the site.

Similarly, displacement of the skull over the sediments within the site is unlikely to account for the perimortem fractures in Cr-17. The sedimentological features of LU-6 (very low-energy depositional environment [21,23]), are incompatible with the high-energy processes necessary to generate such modification *in situ* inside SH. Furthermore, it seems highly improbable that taphonomic processes such as geological transport inside the SH chamber could have produced two episodes of identical blunt force trauma in the same individual, particularly given this singular occurrence among the very large fossil sample recovered from the site.

If the taphonomic processes inside the site are discarded as the cause of the cranial trauma, other possible scenarios can be considered. The location and type of the injury are useful for distinguishing among the potential causes of cranial trauma (i.e. accidental vs violence-related) following forensic criteria [1]. Accidental or unintentional trauma typically affects the sides of the cranial vault, while intentional injuries are more commonly found in the facial region [48–50] (Table 3). Furthermore, falls are usually associated with generalized cranial trauma which tends to produce large linear fractures, especially at the level of the “hat brim line” [44,48–50] (Table 3). Although cranial depression fractures can be a consequence of accidents, they are more likely to be the result of interpersonal violence [2,48]. In the case of Cr-17 it is also possible to rule out the injuries as either self-inflicted or resulting from an unintentional hunting accident, mainly because the lesions involve multiple blows. Based on the absence of cut-marks, other potential post-mortem manipulations (e.g. cannibalism, ritual manipulations, etc.) seem even less likely and more speculative.

**Table 3:** Diagnosis of the trauma according to the mechanism of injury.

Factor			Source
**Severity of the force**	Less force	Linear fractures	[41,44,47]
	More force	Fracture lines radiating from the impact point*	[41,44,47]
**Object characteristics**	Blunt	Radiating fractures are commonly produced with blunt force trauma*; Concentric fractures may present on internal bevel*; Multiple blunt trauma may be indicated by the presence of flakes of bone spalled from fracture margins and the delamination of outer from inner tables*	[39,41,43,44]
	Sharp	Presence of incised wounds; Linear fractures often occur on the skull and result from out-bending of large thin portions of bone as the result of a direct blow of high velocity; Bone fracture lines leading to the sutures.	[35,51,52]
**Cause of the fracture**	Accidental falls	Linear and radial fractures; Lateral location; Fractures on Hat Brim Line^†^; Right side fractures	[1,45,48–50,53,54]
	Aggression	Depressed fractures*; Frontal location*; Fractures above Hat Brim Line * ^†^; Left side fractures*; Multiple blows*	[1,45,48–50,53,54]

*Present in Cr-17 traumatic fractures

†Hat brim line (HBL) corresponds to the area located between two lines parallel to a line inspired by the Frankfurt horizontal plane (horizontal plane passing through right and left porion and the left orbitale), with the superior margin passing through the glabella (G line) and the inferior margin passing through the center of the external auditory meatus (EAM line) [49].

Multiple cranial depression traumas in the frontal region above the hat brim line are compatible with interpersonal violence injuries [1,2,48–50,53–55]. From their consistent size and shape, the Cr-17 blunt force traumas clearly are not unintentional, but, rather, they appear to have been produced by the use of a tool of standardized size and shape. The location of the lesions just to the left of the midline of the frontal squama in Cr-17 is also consistent with the general pattern documented among recent humans, with most individuals showing lesions on the left side of the skull reflecting the predominance of right-handedness during face-to-face conflict [17,56]. Interestingly, the Sima de los Huesos population is considered mainly right-handed [57–59]. The severity of the injuries, with both blows to the head certainly involving penetration of the bone-brain barrier, and the absence of healing via bone remodeling (Fig 3) leads us to consider that this individual did not survive these cranial traumatic events. Indeed, either of the two traumatic events were likely mortal in and of themselves, and the presence of repeated blows might imply a clear intention to kill. Thus, the most plausible explanation for the perimortem fractures on Cr-17 is as the result of intentional and repeated blows during a lethal act of interpersonal violence. This represents the earliest clear case of deliberate, lethal interpersonal aggression in the hominin fossil record, demonstrating that this is an ancient human behavior.

Finally, our results have important implications for the origin of the accumulation of hominin bodies at the SH site. As mentioned previously, geological events and carnivore activity were discarded as the causal agents for the human fossil accumulation [23,25,30]. This leaves only two possible explanations: i) accidental falls of 28 individuals through the vertical shaft, or ii) intentional accumulation of bodies by other humans through the shaft [23].

The present study has established that the individual represented by Cr-17 was already dead before their arrival at the site, and it is possible to rule out an accidental fall as a possible explanation for the arrival of this individual to the SH chamber. The only possible manner by which a deceased individual could have arrived at the SH site is if its cadaver were dropped down the shaft by other hominins. Thus, the interpretation of the SH site as a place where hominins deposited deceased members of their social groups seems to be the most likely scenario to explain the presence of human bodies at the site. This interpretation implies this was a social practice among this group of Middle Pleistocene hominins and may represent the earliest funerary behavior in the human fossil record.

## Supporting Information

S1 Table Craniofacial and postcranial traumatic lesions in the Pleistocene *Homo* fossil record (DOCX)
