# Supplementary material for: Lethal Interpersonal Violence in the Middle Pleistocene
Source: PLoS One. 2015 May 27;10(5):e0126589. doi: 10.1371/journal.pone.0126589 (PMC4446311; doi:10.1371/journal.pone.0126589)
Supplement: S1 Table — (DOCX) [file pone.0126589.s001.docx]

Supporting Information

S1 Table. Craniofacial and postcranial traumatic lesions in the Pleistocene *Homo* fossil record (Updated from Wu et al. [1]).

| **Specimen** | | **Traumatic lesion** | **Source** |
| --- | --- | --- | --- |
| **Early Pleistocene** | |  |  |
|  | Gongwangling/ Lantian 1* | Frontal healed lesion (Trauma or abscess/Postmortem erosion) | [2,3] |
|  | Sangiran 38 | Two parietal depressions possibly post-traumatic | [4] |
| **Middle Pleistocene** | |  |  |
|  | Atapuerca-Trinchera Galería | Parietal healed trauma | [5,6] |
|  | Biache 1 | Shallow exocranial depression | [7] |
|  | Biache 2 | Frontal minor lesion | [7] |
|  | Broken Hill 1 | Temporal trauma with associated infection | [8] |
|  | Casal de’ Pazzi 1 | Depression on the parietal | [9] |
|  | Ceprano 1 | Frontal lesions | [10] |
|  | Ehringsdorf 2 | Parietal lesion | [11] |
|  | Florisbad 1 | Multiple cranial vault and orbital lesions including infection post-trauma | [12] |
|  | Hulu/Nanjing 1 | Healed ectocranial lesion (trauma or burning) | [13] |
|  | La Chaise BD-17 | Parietal shallow depression | [14] |
|  | Maba 1 | Frontal healed trauma (probably interpersonal violence or accident) | [1] |
|  | Ngandong 7 | Parietal depressions | [15] |
|  | Atapuerca-SH-Cranium 1 | Occipital depression | [16] |
|  | Atapuerca-SH-Cranium 2 | Parietal vault depression | [16] |
|  | Atapuerca-SH-Cranium 3 | Parietal depression | [16] |
|  | Atapuerca-SH-Cranium 4 | Frontal and occipital depressions. Parietal depressed groove | [16] |
|  | Atapuerca-SH-Cranium 5 | 12 exocranial lesions, slight supratoral depression and postraumatic maxillary osteitis (traumatic origin) | [16,17] |
|  | Atapuerca-SH-Cranium 6 | Slight exocranial depression | [16] |
|  | Atapuerca-SH-Cranium 7 | Lambdoid suture healed external lesion | [16] |
|  | Atapuerca-SH-Cranium 8 | Slight parietal exocranial depression | [16] |
|  | Atapuerca-SH-Cranium 11 | Frontal healed depression | [16,18] |
|  | Swanscombe 1 | Parietal depressions | [19] |
|  | Zhoukoudian skull X | Frontal and parietal exocranial lesion | [20] |
|  | Zhoukoudian skull XII | Parietal exocranial lesion | [20] |
|  | Zuttiyeh 1 | Two depressed frontal lesions | [21] |
| **Late Pleistocene** | |  |  |
|  | Caviglione 1 | Healed radial fracture | [1] |
|  | Cova Negra (Parietal I) | Trauma with external remodeling | [22,23] |
|  | Dolní Věstonice 3 | Healed traumatic impact on the face | [24] |
|  | Dolní Věstonice 11/12 | Healed depressed fracture of frontal (Possible interhuman violence) | [24] |
|  | Dolní Věstonice 13 | Minor healed frontal trauma | [24] |
|  | Dolní Věstonice 15 | Healed parietal lesion and ulnar fracture | [24] |
|  | Dolní Věstonice 16 | Two external healed frontal lesions | [24] |
|  | Feldhofer 1 | Healed supraorbital arch lesion. Fractured ulna. | [25,26] |
|  | Feldhofer 2 (NN59) | Healed ulnar fracture | [27] |
|  | Kebara KMH2 | Healed fractured thoracic vertebra and metacarpal | [28] |
|  | Kiik-Koba 1 | Healed foot phalanx fracture | [29] |
|  | KNM-LH 1 | Healed frontal trauma | [30] |
|  | Krapina 4 | Healed scalp wound/depression fracture to frontal | [31,32] |
|  | Krapina 20 | Healed lesion in the frontal | [31,32] |
|  | Krapina 31 | Possible healed fracture near temporal line | [31] |
|  | Krapina 34.7 | Healed parietal injury | [31,32] |
|  | Krapina 149 | Healed clavicular fracture | [31,32] |
|  | Krapina 180 | Healed ulnar fracture and pseudoarthrosis | [31,32] |
|  | Krapina 188.8 | Ulnar diaphyseal fracture with osseous callous | [31,32] |
|  | La Chapelle-aux-Saints 1 | Healed fractured rib; generalized osteoarthritis (post-traumatic event) | [33] |
|  | La Ferrassie 1 | Healed displaced greater trochanter | [33,34] |
|  | La Ferrassie 2 | Fibular fracture with infection | [34] |
|  | Le Moustier 1 | Healed mandibular fracture | [35] |
|  | Mladeč 5 | Frontal traumatic lesion | [36] |
|  | Pavlov 1 | Minor healed exocranial alteration | [24] |
|  | Qafzeh 11 | Healed depressed frontal fracture | [37,38] |
|  | Riparo Mezzena | Mandible osteolytic lesion (infection by traumatic fracture) | [39] |
|  | Saint Césaire 1 | Healed frontal trauma (interpersonal violence) | [40] |
|  | Šal’a 1 | Healed frontal depression | [41] |
|  | Shanidar 1 | Facial fracture. Multiple arm fractures with possible amputation. Foot osteoarthritis and metatarsal fracture (accidental or crushing) | [42,43] |
|  | Shanidar 3 | Rib penetrating wound (possible interpersonal violence). Post-traumatic talocrural osteoarthritis | [43,44] |
|  | Shanidar 4 | Healed rib fracture | [43] |
|  | Shanidar 5 | Healed frontal bony scar | [43] |
|  | Skhul 4 | Healed metatarsal fracture | [45] |
|  | Subalyuk 1 | Healed metatarsal fracture | [46] |
|  | Sunghir 1 | Perimortem incision to vertebra T1 (hunting accident or interpersonal violence) | [47,48] |
|  | Sunghir 2 | Possible pelvis perimortem trauma | [48] |
|  | Sunghir 5 | Healed frontal minor lesion | [48] |
|  | Tabun C1 | Diaphyseal lesion on the fibula | [42] |
|  | Xujiayao 5a | Healed parietal injury | [49] |
|  | Xujiayao 8 | Healed parietal injury | [49] |
|  | Xujiayao 12 | Healed occipital injury | [49] |

Five individuals of the Pleistocene hominin record (Maba 1, Dolní Věstonice 11/12, Saint Césaire 1, Shanidar 3 and Sunghir 1) have been interpreted as probable cases of interpersonal violence. The only traumas that have been interpreted as likely representing the cause of death of the individual are those of Sunghir 1 and Shanidar 3. Atapuerca SH-Cranium 5 suffered a chronic infection, probably as a result of an impact, that may have been the cause of death of this individual [17]. Nevertheless, an accidental cause cannot be discarded in any of these cases.

*Caspari [2] identified a lesion in Gongwangling/Lantian 1 due to a trauma or abscess. However, Shang et al. [3] showed that the damage was due to postmortem alteration.

**References**

1. Wu X-J, Schepartz LA, Liu W, Trinkaus E. Antemortem trauma and survival in the late Middle Pleistocene human cranium from Maba, South China. Proc Natl Acad Sci. 2011; 108: 19558-19562.

2. Caspari R. Brief communication: Evidence of pathology on the frontal bone from Gongwangling. Am J Phys Anthropol. 1997; 102: 565-568.

3. Shang H, Trinkaus E, Liu W, Wu X, Zhu Q. Neurocranial abnormalities of the Gongwangling *Homo erectus* from Lantian, China. J Archaeol Sci. 2008; 35: 2589-2593.

4. Indriati E, Antón SC. The calvaria of Sangiran 38, Sendangbusik, Sangiran Dome, Java. Homo. 2010; 61: 225-243.

5. Arsuaga JL, Gracia A, Lorenzo C, Martínez I, Pérez PJ. Resto craneal humano de Galería/Cueva de los Zarpazos (Sierra de Atapuerca, Burgos). In: Carbonell Roura E, Rosas González A, Díez Fernández-Lomana JC, editors. Atapuerca: ocupaciones humanas y paleoecología del yacimiento de Galería: Junta de Castilla y León; 1999. pp. 233–235.

6. Pérez PJ. Recopilación de diagnósticos paleopatológicos en fósiles humanos, con casos relativos a homínidos de Atapuerca. In: Llorens AI, Malgosa Morera A, editors. Paleopatología La enfermedad no escrita. Barcelona: Masson, S.A.; 2003. pp. 295-306.

7. Rougier H. Étude descriptive et comparative de Biache-Saint-Vaast 1 (Biache-Saint-Vaast, Pas-de-Calais, France). Ph.D. Dissertation. 2003; Bordeaux (France): L'Université Bordeaux 1.

8. Montgomery PQ, Williams HOL, Reading N, Stringer CB. An Assessment of the Temporal Bone Lesions of the Broken Hill Cranium. J Archaeol Sci. 1994; 21: 331-337.

9. Manzi G, Salvadei L, Passarello P. The Casal de´Pazzi archaic parietal: comparative analysis of new fossil evidence from the late Middle Pleistocene of Rome. J Hum Evol. 1990; 19: 751-759.

10. Mallegni F, Carnieri E, Bisconti M, Tartarelli G, Ricci S, Biddittu I, et al. *Homo cepranensis* sp. nov. and the evolution of African-European Middle Pleistocene hominids. C R Palevol. 2003; 2: 153-159.

11. Vlček E. Fossile Menschenfunde von Weimar-Ehringsdorf. Ph.D. Dissertation. 1993; Stuttgart.

12. Curnoe D, Brink J. Evidence of pathological conditions in the Florisbad cranium. J Hum Evol. 2010; 59: 504-513.

13. Shang H, Trinkaus E. An ectocranial lesion on the Middle Pleistocene human cranium from Hulu Cave, Nanjing, China. Am J Phys Anthropol. 2008; 135: 431-437.

14. Condemi S. Les Néandertaliens de La Chaise (abri Bourgeois-Delaunay). Paris: Comité des Travaux Historiques et Scientifiques. 2001.

15. Balzeau A, Grimaud-Hervé D, Indriati E, Jacob T. Computer tomography scanning of *Homo erectus* crania Ngandong 7 from Java : Internal structure, paleopathology and post-mortem history. Berkala Llmu Kedokteran. 2003; 35: 133-140.

16. Pérez PJ, Gracia A, Martínez I, Arsuaga JL. Paleopathological evidence of the cranial remains from the Sima de los Huesos Middle Pleistocene site (sierra de Atapuerca, Spain). Description and preliminary inferences. J Hum Evol. 1997; 33: 409-421.

17. Gracia-Téllez A, Arsuaga JL, Martínez I, Martín-Frances L, Martinón-Torres M, Bermúdez de Castro JM, et al. Orofacial pathology in *Homo heidelbergensis*: the case of Skull 5 from the Sima de los Huesos site (Atapuerca, Spain). Quat Int. 2013; 295: 83-93.

18. Arsuaga JL, Martínez I, Arnold LJ, Aranburu A, Gracia A, Sharp WD, et al. Neandertal roots: Cranial and chronological evidence from Sima de los Huesos. Science. 2014; 344: 1358-1363.

19. Le Gros Clark WE. General features of the Swanscombe skull bones. J R Anthropol Inst. 1938; 68: 58-67.

20. Weidenreich F. The skull of *Sinanthropus pekinensis*; a comparative study on a primitive hominid skull. Palaeontol Sínica. 1943; 10D: 1-485.

21. Simmons T, Falsetti AB, Smith FH. Frontal bone morphometrics of southwest Asian Pleistocene hominids. J Hum Evol. 1991; 20: 249-269.

22. Fusté M. Parietal Neandertalense de Cova Negra (Játiva). Servicio de Investigación Prehistórica. 1953; 17: 1-36.

23. Lumley MA. Anteneandertaliens et Neandertaliens du basin Mediterraneen Occidental Europeen. Marseille: Laboratoire de paleontologie humaine et de prehistoire. Universite de Provence. 1973.

24. Trinkaus E, Hillson SW, Franciscus RG, Holliday TW. Skeletal and dental paleopathology. In: Trinkaus E, Svoboda JA, editors. Early Modern human evolution in Central Europe The people of Dolní Věstonice and Pavlov. New York: Oxford University Press; 2006. pp. 419-458.

25. Schultz M. Results of the anatomical-palaeopathological investigations on the Neanderthal skeleton from Klein Feldhofer Grotte (1856) including the new discoveries from 1997/2000. In: Schmitz RW, editor. Neanderthal 1856-2006: Verlag Philipp von Zabern; 2006. pp. 278-318.

26. Trinkaus E, Churchill SE, Ruff CB. Postcranial robusticity in *Homo*. II: Humeral bilateral asymmetry and bone plasticity. Am J Phys Anthropol. 1994; 93: 1-34.

27. Smith FH, Ostendorf Smith M, Schmitz RW. Human skeletal remains from the 1997 and 2000 excavations of cave deposits derived from Kleine Feldhofer Grotte in the neander valley, Germany. In: Schmitz RW, editor. Neanderthal 1856-2006: Verlag Philipp von Zabern; 2006. pp. 187-246.

28. Duday H, Arensburg B. La pathologie. In: Bar-Yosef O, Vandermeersch B, editors. Le Squelette Moustérien de Kébara 2. Paris: CNRS; 1991. pp. 179–193.

29. Trinkaus E, Maley B, Buzhilova AP. Paleopathology of the Kiik-Koba 1 Neandertal. Am J Phys Anthropol. 2008; 137: 106–112.

30. Tryon CA, Crevecoeur I, Faith JT, Ekshtain R, Nivens J, Patterson D, et al. Late Pleistocene age and archaeological context for the hominin calvaria from GvJm-22 (Lukenya Hill, Kenya). Proc Natl Acad Sci. 2015; doi:10.1073/pnas.1417909112.

31. Gardner JC, Smith FH. The Paleopathology of the Krapina Neandertals. Periodicum Biol. 2006; 108: 471-484.

32. Radovčić J, Smith FH, Trinkaus E, Wolpoff MH. The Krapina Hominids. An illustrated catalog of skeletal collection. Mladost, Zagreb: Croatian Natural History Museum. 1988.

33. Trinkaus E. Pathology and the posture of the La Chapelle-aux-Saints Neandertal. Am J Phys Anthropol. 1985; 67: 19-41.

34. Heim JL. Les Hommes Fossiles de La Ferrassie. Tome II. Les Squelettes adultes (squelette des membres). Paris: Masson. 1982.

35. Ponce de León MS, Zollikofer CPE. New evidence from Le Moustier 1: Computer-assisted reconstruction and morphometry of the skull. Anat Rec. 1999; 254: 474-489.

36. Teschler-Nicola M, Czerny C, Oliva M, Schamall D, Schultz M. Pathological alterations and traumas in the human skeletal remains from Mladeč. In: Teschler-Nicola M, editor. Early Modern Humans at the Moravian Gate The Mladeč Caves and their remains. New York: Springer Vienna; 2006. pp. 473-487.

37. Coqueugniot H, Dutour O, Arensburg B, Duday H, Vandermeersch B, Tillier A-m. Earliest cranio-encephalic trauma from the levantine Middle Palaeolithic: 3D reappraisal of the Qafzeh 11 Skull, consequences of pediatric brain damage on individual life condition and social care. PLoS ONE. 2014; 9: e102822.

38. Tillier AM. Les enfants moustériens de Qafzeh. Interprétation phylogénétique et paléoauxologique. Paris: Cahiers de Paléoanthropologie, CNRS Éditions. 1999.

39. Condemi S, Tardivo D, Foti B, Ricci S, Giunti P, Longo L. A case of an osteolytic lesion on an Italian Neanderthal jaw. C R Palevol. 2012; 11: 79-83.

40. Zollikofer CPE, Ponce de León M, Vandermeersch B, Leveque F. Evidence for interpersonal violence in the St. Cesaire Neanderthal. Proc Natl Acad Sci. 2002; 99: 6444-6448.

41. Sládek V, Trinkaus E, Sefcáková A, Halouzka R. Morphological affinities of the Sal’a 1 frontal bone. J Hum Evol. 2002; 43: 787-815.

42. Berger TD, Trinkaus E. Patterns of trauma among the Neandertals. J Archaeol Sci. 1995; 22: 841-852.

43. Trinkaus E. The Shanidar Neandertals. New York: Academic Press. 1983.

44. Churchill SE, Franciscus RG, McKean-Peraza HA, Daniel JA, Warren BR. Shanidar 3 Neandertal rib puncture wound and paleolithic weaponry. J Hum Evol. 2009; 57: 163-178.

45. McCown TD, Keith A. The Stone Age of Mount Carmel. The Fossil Human Remains from the Levalloiso-Mousterian. Oxford: Clarendon Press; 1939. pp.

46. Pap I, Tillier AM, Arensburg B, Chech M. The Subalyuk Neanderthal remains (Hungary): a reexamination. Ann Hist Nat Mus Nationalis Hungarici. 1996; 88: 253-270.

47. Trinkaus E, Buzhilova AP. The death and burial of Sunghir 1. Int J Osteoarchaeol. 2012; 22: 655-666.

48. Trinkaus E, Buzhilova AP, Mednikova MB, Dobrovolskaya MV. The People of Sunghir: Burials, Bodies, and Behavior in the Earlier Upper Paleolithic. New York: Oxford University Press. 2014.

49. Wu JH, Trinkaus E. Neurocranial trauma in the Late Archaic human remains from Xujiayao, Northern China. Int J Osteoarchaeol. In press; doi: 10.1002/oa.2283.
